# Nasal Colonization of Antimicrobial‐Resistant and Biofilm‐Producing *Staphylococcus* spp. in Surgical Canine Patients From Italy

**DOI:** 10.1002/vms3.71235

**Published:** 2026-09-23

**Authors:** Francesca Paola Nocera, Rossana Schena, Annunziata Romano, Stefano Cavalli, Laura Cortese, Gerardo Fatone, Luisa De Martino

**Affiliations:** ^1^ Department of Veterinary Medicine and Animal Production University of Naples Federico II Naples Italy

**Keywords:** biofilm formation, multidrug resistance, nasal colonization, *Staphylococcus pseudintermedius*, surgical canine patients

## Abstract

**Background:**

*Staphylococcus* spp. commonly colonise the skin and mucosal surfaces of dogs and may act as opportunistic pathogens under favourable conditions. Antimicrobial resistance and biofilm formation are key traits that may contribute to bacterial persistence and reduced susceptibility to antimicrobial agents.

**Objectives:**

This study aimed to characterise the antimicrobial resistance profiles and biofilm‐forming ability of *Staphylococcus* spp. isolated from the nasal cavities of dogs admitted to the Surgical Unit of the Veterinary Teaching Hospital in Naples, Italy.

**Methods:**

Nasal swabs were collected from 100 dogs prior to surgery. Strains were identified by MALDI‐TOF mass spectrometry. Antimicrobial susceptibility testing was performed using the disk diffusion method according to international guidelines. Biofilm formation was assessed using a quantitative crystal violet assay.

**Results and Conclusions:**

A total of 65 *Staphylococcus* strains were recovered. *Staphylococcus pseudintermedius* was the most prevalent species (40%; χ^2^ = 134.6, *df* = 14, *p* < 0.001), followed by *Staphylococcus aureus* (15.4%) and *Staphylococcus epidermidis* (7.7%). High levels of resistance were observed to several antimicrobials, with 61.5% and 4.6% of strains being classified as multidrug‐resistant (MDR) and extensively drug‐resistant (XDR), respectively. Notably, all tested isolates exhibited biofilm formation, with the majority (56.9%) classified as moderate producers.

These findings highlight the presence of antimicrobial‐resistant and biofilm‐forming *Staphylococcus* spp. in the nasal cavities of dogs undergoing surgery. The coexistence of these traits may contribute to bacterial persistence, representing a concern for clinical management. Therefore, preoperative nasal screening in high‐risk patients should be considered to optimize prophylactic antimicrobial protocols and reinforce infection control in veterinary surgical settings.

## Graphical abstract

Nasal Staphylococci in Surgical Canine Patients

This study highlights a high occurrence of multidrug‐resistant (MDR) staphylococci isolated from nasal swabs of canine surgical patients, with *Staphylococcus pseudintermedius* being the most frequent pathogen. Crucially, the majority of these strains demonstrated moderate‐to‐strong biofilm‐forming abilities, which occurred independently of their antimicrobial resistance profiles.

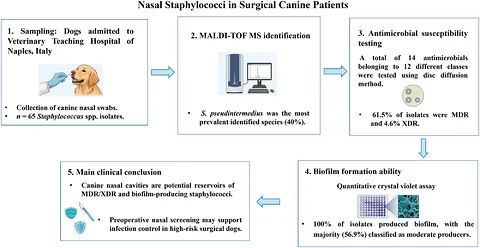

## 1 Introduction

*Staphylococcus* species are among the most common bacterial inhabitants of the skin and mucosal surfaces of humans and animals (Parlet et al. 2019; Wesołowska and Szczuka 2023; Bertelloni et al. 2021; Nocera et al. 2023). These microorganisms are often considered part of the normal microbiota but may act as opportunistic pathogens when host defences are compromised or when bacterial virulence factors facilitate colonization and persistence. In companion animals, several *Staphylococcus* species have been identified as common colonizers of the skin, nasal cavity and other mucosal sites (Weese 2013).

Among these species, *Staphylococcus pseudintermedius* (*S. pseudintermedius)* is regarded as the predominant staphylococcal species associated with dogs, and represents an important opportunistic pathogen responsible for a wide range of infections, including skin and soft tissue infections, otitis and postoperative wound infections. Other species, such as *Staphylococcus aureus* (*S. aureus*) and coagulase‐negative staphylococci, may also be present in the canine microbiota and occasionally contribute to disease under predisposing clinical conditions (Nocera et al. 2023; Fazakerley et al. 2009; Nocera, Meroni et al. 2020).

In recent years, antimicrobial resistance among staphylococci isolated from companion animals has become an increasing concern in veterinary medicine. Methicillin‐resistant *S. pseudintermedius* (MRSP) has emerged worldwide and is often associated with multidrug‐resistant phenotypes that may complicate therapeutic management (Nocera et al. 2021; Miszczak et al. 2023). Continuous monitoring of antimicrobial resistance in staphylococcal populations from companion animals is therefore important to better understand the distribution of resistant strains and support appropriate antimicrobial use in veterinary practice.

Another bacterial trait that may contribute to persistence and reduced susceptibility to antimicrobial treatment is the ability to form biofilms. Biofilms are structured microbial communities embedded in a self‐produced extracellular matrix that allows bacteria to adhere to surfaces and survive under adverse conditions. Within these structures, bacterial cells may exhibit altered metabolic activity and increased tolerance to antimicrobial agents and host immune responses. Biofilm formation has been described in several staphylococcal species isolated from animals and is considered a potential virulence‐associated characteristic (Ruiz‐Romero and Vargas‐Bello‐Pérez 2023). The nasal cavity constitutes an important ecological niche for staphylococci and acts as a key reservoir for strains harbouring antimicrobial resistance and virulence‐associated traits (Abdullahi et al. 2021). Studies investigating the microbiological characteristics of nasal staphylococci in dogs remain relatively limited, particularly in animals admitted to veterinary hospitals for surgical procedures.

Therefore, the aim of the present study was to characterise *Staphylococcus* spp. isolated from the nasal cavities of dogs undergoing surgical procedures at the Veterinary Teaching Hospital in Naples, Italy, with particular focus on antimicrobial resistance profiles and biofilm‐forming ability, as well as to explore potential associations between resistance profiles and biofilm production.

## 2 Materials and Methods

### 2.1 Ethical Statement and Consent to Participate

This study was approved by the Institutional Animal Welfare Committee (OPBA, CSV, University of Naples Federico II, PG/2023/0120379 del 05/10/2023) in compliance with the Italian Legislative Decree 26/2014, Article 2, implementing Directive 2010/63/EU.

Nasal swab samples were collected from household dogs undergoing orthopaedic surgery at the Veterinary Teaching Hospitals of the Department of Veterinary Medicine and Animal Production, University of Naples Federico II. All samples were obtained preoperatively by licensed veterinarians as part of routine clinical procedures, ensuring minimal discomfort and no additional risk to the animals. No invasive procedures were performed, and sample collection did not entail any additional risk beyond standard clinical handling. Written informed consent was obtained from the owners of the dogs prior to enrolment in the study.

### 2.2 Sampling

In this study, dogs were enrolled upon admission for surgical procedures. Non‐invasive nasal swabs were collected during the preoperative phase, primarily from patients undergoing orthopaedic surgery. To minimize animal stress, swabs were obtained while the patients were under general anaesthesia. For each patient, a single swab was inserted approximately 2–3 cm deep into both nostrils, rotated 360° twice, and then placed into Stuart transport medium (Aptaca Spa, Asti, Italy). Specimens were transferred to the BLaboratory of Microbiology of the Department of Veterinary Medicine and Animal Production (University of Naples Federico II, Italy) within 2 h of collection for bacteriological investigation.

### 2.3 Isolation and Identification of *Staphylococcus* spp

Canine nasal swabs were processed at the Microbiology Laboratory, Department of Veterinary Medicine and Animal Production, University of Naples Federico II. For *Staphylococcus* spp. isolation, samples were streaked onto Mannitol salt agar (MSA) and Columbia CNA agar with 5% sheep blood (Liofilchem, Teramo, Italy), then incubated aerobically at 37°C for 24 h.

Presumptive *Staphylococcus* spp. colonies were assessed for morphology, mannitol fermentation and haemolysis, followed by Gram staining. Catalase (Biomérieux, Marcy‐l’Étoile, France) and coagulase tests (Liofilchem, Teramo, Italy) were performed to differentiate coagulase‐positive and ‐negative isolates.

Distinct colonies were subcultured on CNA agar to obtain pure isolates and identified by matrix‐assisted laser desorption ionization time‐of‐flight mass spectrometry (MALDI‐TOF MS; MALDI Biotyper Sirius_Bruker Daltonics, Bremen, Germany). Single colonies were spotted in triplicate onto 96‐spot MBT Biotargets, overlaid with 1 µL α‐cyano‐4‐hydroxycinnamic acid matrix, air‐dried, and analysed using FlexControl 3.4 software. Bruker's Bacterial Test Standard (BTS) was used for calibration. Identification scores were interpreted as: ≥ 2.0 (species level), 1.70–1.99 (genus level), <1.70 (no identification).

*S. aureus* ATCC 33591 and *S. pseudintermedius* ATCC 49444 served as quality control strains. Pure isolates were stored in tryptone soy broth with 30% glycerol at –20°C for further analyses.

### 2.4 Antimicrobial Susceptibility Testing and Phenotypic Profiling

The phenotypic antimicrobial resistance profiles of the isolated and identified *Staphylococcus* spp. strains were determined using the disk diffusion method on Mueller–Hinton agar plates (Liofilchem, Teramo, Italy). A total of 14 antimicrobials belonging to 12 different classes were tested, selected based on their relevance and frequent use in small animal veterinary clinical practice: penicillin (P, 10 IU), oxacillin (OX, 1 µg), cefoxitin (FOX, 30 µg), fusidic acid (FA, 10 µg), rifampicin (RD, 30 µg), ciprofloxacin (CIP, 5 µg), clindamycin (CD, 2 µg), erythromycin (E, 15 µg), enrofloxacin (ENR, 5 µg), gentamicin (CN, 10 µg), chloramphenicol (C, 30 µg), sulfamethoxazole‐trimethoprim (SXT, 23.75/1.25 µg), tetracycline (TE, 30 µg) and linezolid (LNZ, 30 µg). Antimicrobial susceptibility results were interpreted according to the *Staphylococcus* spp. breakpoints established by the European Committee on Antimicrobial Susceptibility Testing (EUCAST) (EUCAST 2025). Enrofloxacin, a veterinary antimicrobial agent, was interpreted using animal‐specific criteria provided by the Clinical and Laboratory Standards Institute (CLSI) (CLSI, 2020).

Methicillin resistance was determined using disk diffusion testing and interpreted as a phenotypic surrogate marker according to EUCAST guidelines (EUCAST 2025). Cefoxitin disk diffusion was used for *S. aureus* and coagulase‐negative staphylococci, whereas oxacillin was used for *S. pseudintermedius* and *S. intermedius*. Molecular detection of *mecA* and *mecC* genes was not performed for the genotypic characterization of methicillin resistance.

Isolated strains were categorised according to the antimicrobial resistance definitions standardized by Magiorakos et al. (2012). For this classification, full resistance to at least one representative agent within an antimicrobial class was used to define resistance for that entire category. Specifically, strains were classified as multidrug‐resistant (MDR) if they exhibited resistance to at least one agent in three or more antimicrobial categories, extensively drug‐resistant (XDR) if resistant to at least one agent in all but two or fewer categories, and pandrug‐resistant (PDR) if they demonstrated resistance to all agents across all categories tested.

### 2.5 Assessment of Biofilm‐Forming Ability Among Isolated Strains

The biofilm‐forming ability of *Staphylococcus* spp. strains was assessed in vitro using the quantitative crystal violet assay, following the method described by Stepanovic et al. (2007), with minor modifications as previously reported by Nocera et al. (2025). Overnight cultures of isolated staphylococci were adjusted to an OD_600_ of 0.2 (≈10^8^ CFU/mL) using fresh brain heart infusion (BHI) broth (Liofilchem, Teramo, Italy). The standardised suspensions were then diluted 1:2 in 200 µL of BHI broth and dispensed into sterile, flat‐bottomed 96‐well polystyrene microplates (Corning Inc., New York, USA). Control wells contained 200 µL of BHI broth alone, which served as the negative control to verify medium sterility and establish background OD levels. Microplates were incubated at 37°C for 24 h, after which the contents were discarded, and the wells were gently washed three times with sterile phosphate‐buffered saline (PBS) to remove non‐adherent planktonic cells and subsequently air‐dried. The adherent biofilms were stained with 200 µL of 1% crystal violet (BioMérieux, Marcy l'Etoile, France) for 30 min at room temperature. Excess dye was removed by washing with distilled water and bound crystal violet was solubilized with 150 µL of 100% ethanol for 15 min at room temperature. The absorbance of the resulting stained biofilms was measured at 570 nm using a microplate reader (Multiskan FC, Thermo Fisher Scientific, Milan, Italy). All assays were carried out in triplicate and repeated three independent times.

In accordance with Stepanovic et al. (2007), biofilm‐forming ability was determined by comparing the mean OD of the strains with that of the negative control and the cut‐off value (ODc). Strains were classified as follows:

*No biofilm formation*: OD ≤ ODc
*Weak biofilm formation*: ODc < OD ≤ 2 × ODc
*Moderate biofilm formation*: 2 × ODc < OD ≤ 4 × ODc
*Strong biofilm formation*: OD > 4× ODc.

### 2.6 Statistical Analysis

All bacteriological results were systematically recorded in a standardized format and entered into Microsoft 365 Excel (Microsoft Corp., Redmond, WA, USA) for data management and analysis. Descriptive statistical analyses were performed by calculating absolute frequencies, percentages and 95% confidence intervals (95% CI) to determine the occurrence, phenotypic antimicrobial resistance profiles, and biofilm formation ability of *Staphylococcus* spp. strains. Graphical representations were also generated within the same software. Inferential statistical analyses were performed using JASP (Version 0.96.0; JASP Team 2026, Amsterdam, the Netherlands). Specifically, a Chi‐square goodness‐of‐fit test was used to determine whether *S. pseudintermedius* was the most frequently isolated species with statistical significance. To evaluate the relationship between bacterial species (categorized as *S. pseudintermedius* vs. other *Staphylococcus* spp.) and their antimicrobial resistance profiles (MDR/XDR vs. non‐MDR), Fisher's exact test was employed. Similarly, within the *S. pseudintermedius* subset, Pearson's Chi‐square test was applied to assess the association between resistance categories (MDR/XDR vs. non‐MDR) and biofilm production capacity, classified as weak, moderate or strong. For all analyses, a *p*‐value < 0.05 was considered statistically significant.

## 3 Results

### 3.1 Recovery and Identification of Staphylococci

Of the 100 canine nasal swabs collected from both nostrils of individual dogs, 36% yielded no bacterial growth, while 64% were positive for bacterial growth. Of these, staphylococcal strains were successfully isolated and identified using proteomic profiling by MALDI‐TOF MS. All strains showed reliable species‐level identification with log(scores) ≥ 2.00.

**FIGURE 1:**
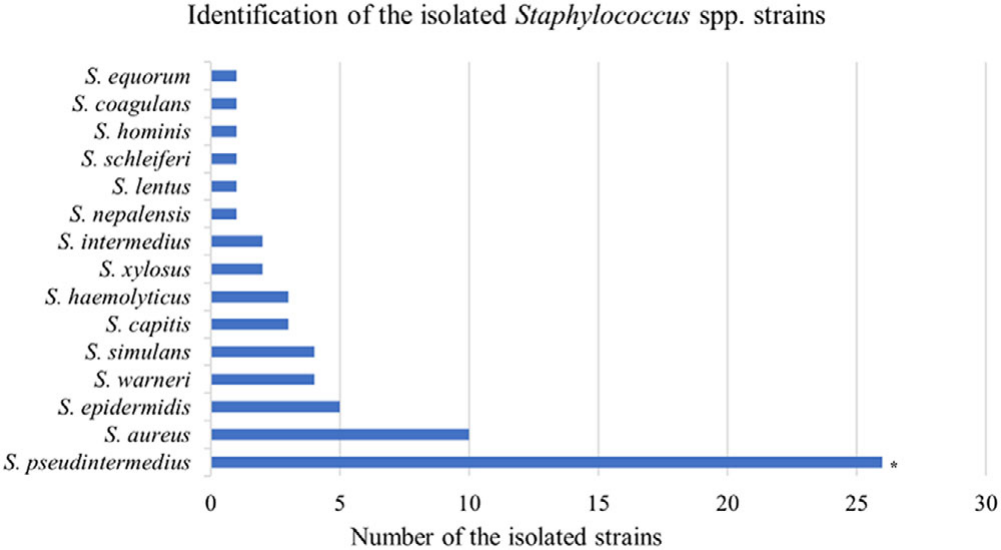
*Staphylococcus* spp. strains identified from canine nasal swabs. Bars represent the total number of isolated strains for each species. The asterisk (*) denotes that *S. pseudintermedius* was the significantly predominant species compared to the others, as determined by a multinomial Chi‐square test (χ^2^ = 134.6, *df* = 14, *p* < 0.001).

Among the staphylococci isolated from all samples, *S. pseudintermedius* was the most prevalent species (40%; 26/65; 95% CI: 28.0%–52.9%), and it was significantly more frequent compared with the other species (χ^2^ = 134.6, *df* = 14, *p* < 0.001). This was followed by *S. aureus* (15.4%; 10/65; 95% CI: 7.6%–26.5%), *S. epidermidis* (7.7%; 5/65; 95% CI: 2.5%–17.0%), and *S. simulans* and *S. warneri*, both with a frequency of 6.2% (4/65; 95% CI: 2.0%–15.0%) (Figure 1). Other *Staphylococcus* species were also isolated at lower frequencies (Figure 1). Notably, from one surgical canine patient, a co‐isolation of both *S. haemolyticus* and *S. equorum* was successfully recovered (ID 28).

### 3.2 Antimicrobial Susceptibility Profiles of Recovered *Staphylococcus* spp. strains

**FIGURE 2:**
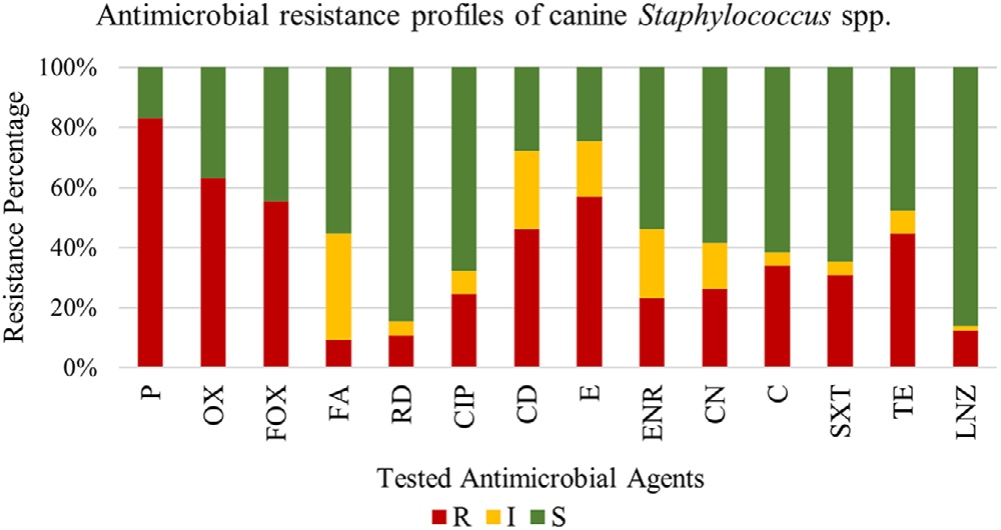
Antimicrobial resistance profiles of isolated canine *Staphylococcus* spp. strains. C, chloramphenicol; CD, clindamycin; CIP, ciprofloxacin; CN, gentamicin, E, erythromycin; ENR, enrofloxacin; FA, fusidic acid; FOX, cefoxitin; LNZ, linezolid; OX, oxacillin; P, penicillin; RD, rifampicin; SXT, sulfamethoxazole‐trimethoprim; TE, tetracycline.

Antimicrobial susceptibility testing revealed a high prevalence of antimicrobial resistance among strains, particularly to penicillin (83.1%; 54/65; 95% CI: 72.3%–90.5%), oxacillin (63.1%; 41/65; 95% CI: 50.9%–73.9%) and cefoxitin (55.4%; 36/65; 95% CI: 43.3%–66.9%) (Figure 2). High levels of resistance were also recorded for erythromycin (56.9%; 37/65; 95% CI: 44.0%–69.2%), clindamycin (46.2%; 30/65; 95% CI: 34.0%–58.7%) and tetracycline (44.6%; 29/65; 95% CI: 32.6%–57.1%). Lower resistance rates (< 20%) were observed for fusidic acid (9.2%; 6/65; 95% CI: 4.3%–18.7%), rifampicin (10.8%; 7/65; 95% CI: 5.3%–20.7%) and linezolid (12.3%; 8/65; 95% CI: 6.4%–22.5%).

**FIGURE 3:**
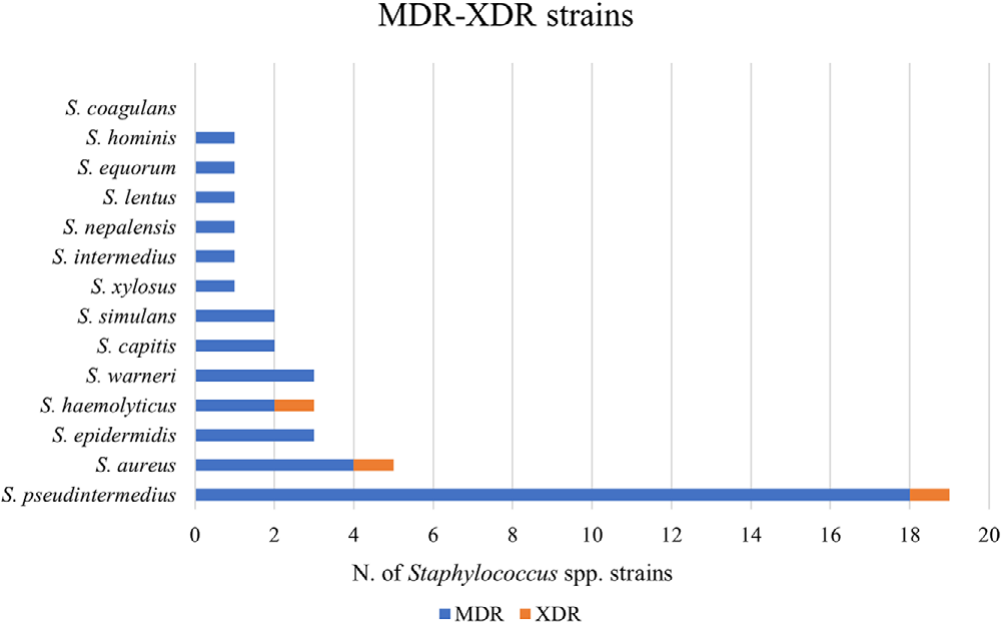
MDR and XDR *Staphylococcus* spp. strains from canine nasal cavities.

Overall, 40 out of 65 strains (61.5%; 95% CI: 49.3%–72.6%) exhibited MDR profiles, while 3/65 (4.6%; 95% CI: 1.6%–12.7%) were classified as XDR (Figure 3), resulting in a cumulative MDR/XDR prevalence of 66.2% (43/65). Of note, the wide confidence intervals observed for low‐frequency estimates (such as XDR) reflect the limited sample size and indicate reduced precision, warranting cautious interpretation. No PDR strains were identified.

Specifically, among *S. pseudintermedius* strains (*n* = 26), 18/26 were MDR (69.2%; 95% CI: 48.2%–85.7%) and 1/26 was XDR (3.8%; 95% CI: 0.1%–19.6%), representing a combined MDR/XDR rate of 73.1% (19/26). Although this resistance rate was higher than that observed in the other *Staphylococcus* species combined (61.5%; 24/39), Fisher's exact test showed that the difference was not statistically significant (*p* = 0.426).

### 3.3 Determination of Biofilm‐Forming Ability

**FIGURE 4:**
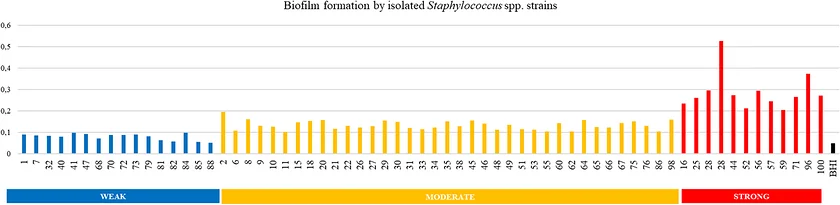
Biofilm forming ability of canine *Staphylococcus* spp. Biofilm production was assessed using the quantitative crystal violet assay, and strains were categorized as non‐biofilm producers, weak, moderate or strong producers according to the criteria described in the Materials and Methods section. The mean OD_570 nm_ of the negative control, used to determine ODc, was 0.05.

The staphylococcal strains were evaluated for biofilm‐forming ability (Figure 4). Among all isolates, 16/65 (24.6%; 95% CI: 15.3%–36.1%) were classified as weak biofilm producers, 37/65 (56.9%; 95% CI: 44.0%–69.2%) as moderate producers, and 12/65 (18.5%; 95% CI: 9.9%–30.0%) as strong producers. No strains without biofilm production were observed (0/65; 95% CI: 0.0%–5.5%).

**FIGURE 5:**
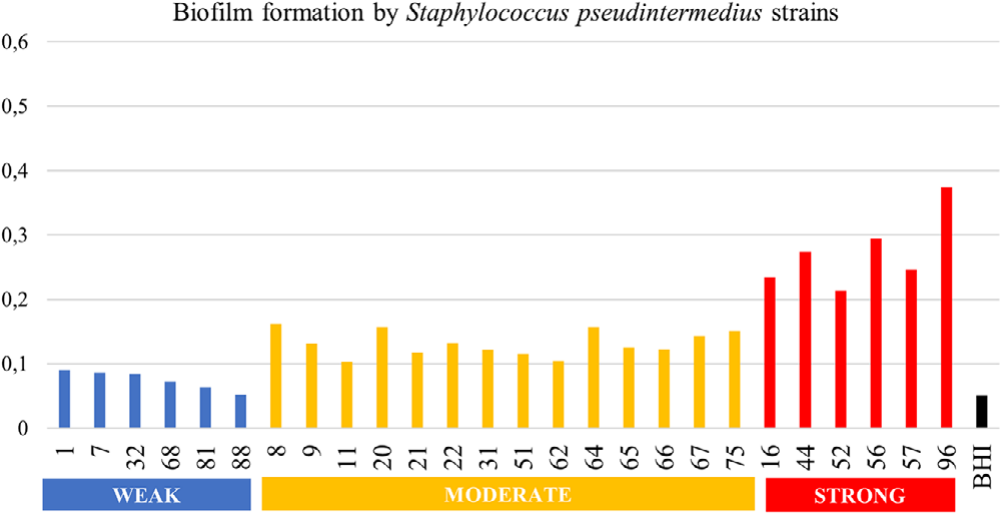
Biofilm forming ability of investigated *S. pseudintermedius* strain from dogs. Biofilm production was assessed using the quantitative crystal violet assay, and strains were categorized as non‐biofilm producers, weak, moderate, or strong producers according to the criteria described in the Materials and Methods section. The mean OD_570 nm_ of the negative control, used to determine ODc, was 0.05.

Particular attention was given to *S. pseudintermedius* due to its high isolation frequency. Among the 26 isolates, 6/26 (23.1%; 95% CI: 9.0%–43.6%) were weak biofilm producers, 14/26 (53.8%; 95% CI: 33.4%–73.4%) were moderate producers and 6/26 (23.1%; 95% CI: 9.0%–43.6%) were strong producers (Figure 5). Notably, one strong biofilm‐producing strain was susceptible to all tested antimicrobials.

**FIGURE 6:**
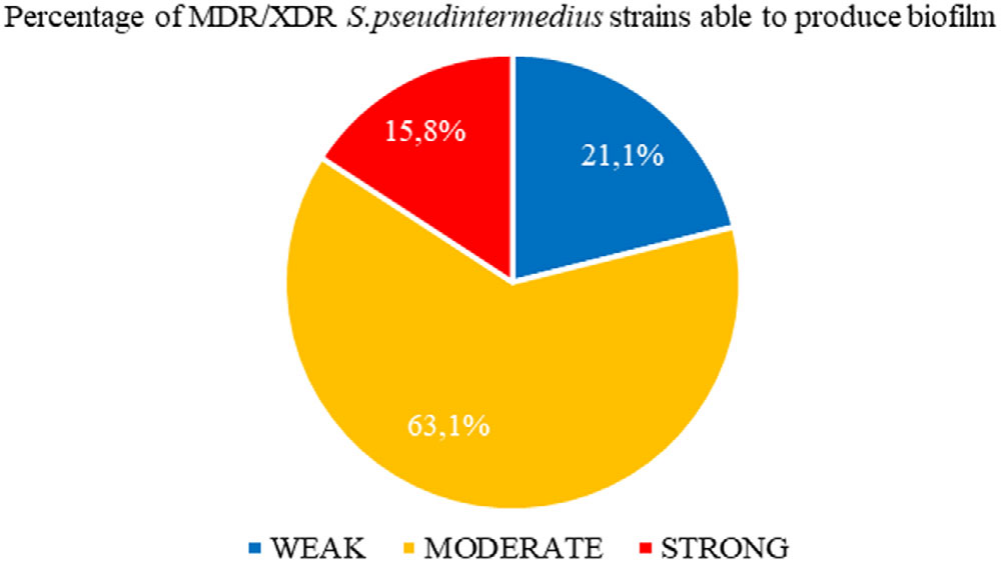
Correlation between biofilm‐producing phenotypes and the MDR/XDR profiles of canine *S. pseudintermedius* strains. Statistical analysis (Pearson's Chi‐square test) revealed no significant association between antimicrobial resistance profiles and biofilm forming ability (χ^2^ = 2.886, *df* = 2, *p* = 0.236).

When analysing the distribution of biofilm‐forming phenotypes in relation to antimicrobial resistance profiles of *S. pseudintermedius* strains (*n* = 19; 18 MDR, 1 XDR), 4/19 (21.1%; 95% CI: 6.1%–45.6%), 12/19 (63.1%; 95% CI: 38.4%–83.7%), and 3/19 (15.8%; 95% CI: 3.4%–39.6%) were classified as weak, moderate and strong biofilm producers, respectively (Figure 6). The XDR strain was classified as a moderate biofilm producer. In comparison, among the 7 non‐MDR strains, 2/7 (28.6%; 95% CI: 3.7%–71.0%) were weak, 2/7 (28.6%; 95% CI: 3.7%–71.0%) were moderate and 3/7 (42.9%; 95% CI: 9.9%–81.6%) were strong biofilm producers. Pearson's Chi‐square test revealed no statistically significant association between the multidrug resistance profile and biofilm production ability (χ^2^ = 2.886, *df* = 2, *p* = 0.236), indicating that biofilm‐forming ability occurs independently of the antimicrobial resistance status in this subset. The specific cross‐distribution of phenotypic resistance profiles (MDR/XDR) and biofilm formation categories for *S. pseudintermedius* and the other *Staphylococcus* species is systematically summarised in Table 1.

**TABLE 1:** Cross‐distribution of multidrug resistance (MDR/XDR) and biofilm formation categories between *S. pseudintermedius* and other *Staphylococcus* spp.

Bacterial group	MDR (%) *n*	XDR (%) *n*	Weak biofilm producers (%) *n*	Moderate biofilm producers (%) *n*	Strong biofilm producers (%) *n*
*S. pseudintermedius*	69.2% (18/26)	3.8% (1/26)	23.1% (6/26)	53.8% (14/26)	23.1% (6/26)
Other *Staphylococcus* spp.	56.4% (22/39)	5.1% (2/39)	25.6% (10/39)	59% (23/39)	15.4% (6/39)
Total	61.5% (40/65)	4.6% (3/65)	24.6% (16/65)	56.9% (37/65)	18.5% (12/65)

*Note*: 95% confidence intervals are provided in Section 3 for the relevant estimates.

## 4 Discussion

Staphylococcal nasal colonization represents a key ecological niche for opportunistic pathogens in both humans and animals (Weese and van Duijkeren 2010; Windahl et al. 2012). Within the canine skin and mucosal microbiota, *S. pseudintermedius* is a prominent commensal (Weese 2013; Weese and van Duijkeren 2010) that can cause skin, soft tissue, wound and postoperative infections (Nazarali et al. 2015; Singh et al. 2013; Viegas et al. 2022; Feuer et al. 2024; Kim et al. 2025; Nocera and De Martino 2024). The clinical impact of this opportunistic pathogen is significantly amplified when it exhibits enhanced virulence traits, such as multidrug resistance and biofilm production (Nocera, Meroni, et al. 2020; Singh et al. 2013; Viegas et al. 2022; Feuer et al. 2024; Kim et al. 2025; Nocera and De Martino 2024).

In this study, 65 *Staphylococcus* spp. strains were isolated from the nasal cavities of dogs admitted for surgery at the Veterinary Teaching Hospital in Naples. *S. pseudintermedius* was the most prevalent species, followed by *S. aureus*, *S. epidermidis*, *S. simulans* and *S. warneri*, confirming the diversity of staphylococcal populations inhabiting this anatomical site (Nocera et al. 2023; Becker et al. 2014).

Antimicrobial susceptibility testing revealed a relatively high prevalence of resistance to several agents, including penicillin, oxacillin, cefoxitin, erythromycin, clindamycin and tetracycline, consistent with the increasing trend in antimicrobial resistance among canine staphylococci (Windahl et al. 2012; Singh et al. 2013; Viegas et al. 2022; Feuer et al. 2024; Kim et al. 2025). Given the lack of genotypic confirmation through *mecA*/*mecC* PCR, these oxacillin and cefoxitin resistance profiles must be interpreted as phenotypic indicators of methicillin resistance, as alternative resistance mechanisms (e.g., β‐lactamase hyperproduction) cannot be completely ruled out. Notably, the high detection rate of MDR strains (61.5%; 40/65; 95% CI: 49.3%–72.6%) underscores substantial selective pressure in companion animals and highlights the growing challenge in clinical management, emphasizing the need for continuous surveillance in veterinary clinical settings (Kadlec and Schwarz 2012; Nocera, Parisi, et al. 2020; Nocera et al. 2025).

Another key aspect investigated was the biofilm‐forming ability, a phenotype that promotes bacterial persistence on mucosal surfaces, environmental survival, and increased tolerance to antimicrobials, potentially driving nosocomial transmission (Osland et al. 2012; Costerton et al. 1999; Oliveira and Cunha Mde 2010). All isolates in this study exhibited biofilm production, with the majority classified as moderate producers (56.9%; 95% CI: 44.0%–69.2%). These results agree with previous studies showing that biofilm formation is common among canine staphylococci, although the level of production varies, with moderate‐to‐strong phenotypes frequently observed in clinical isolates (Singh et al. 2013; Arima et al. 2018; Silva et al. 2022). Although the clinical significance of biofilm production in nasal colonization is not fully understood, this phenotype may enhance bacterial persistence on mucosal surfaces and facilitate survival under adverse conditions. In addition, it may contribute to chronic opportunistic infections and promote environmental persistence within the hospital facilities (Osland et al. 2012; Ziebuhr et al. 2006).

Given its clinical relevance, a species‐specific focus was directed at *S. pseudintermedius*, as it represented the most frequently isolated species in this study (40%; χ^2^ = 134.6, *df* = 14, *p* < 0.001). Within this group, moderate biofilm producers predominated (53.8%; 14/26; 95% CI: 33.4%–73.4%), while a considerable share exhibited MDR profiles (69.2%; 18/26; 95% CI: 48.2%–85.7%), and only one showed an XDR profile (3.8%; 1/26; 95% CI: 0.1%–19.6%). Although phenotypic analysis demonstrated that biofilm formation capacity occurs independently of antimicrobial resistance (χ^2^ = 2.886, *df* = 2, *p* = 0.236), the coexistence of these traits remains a concern for bacterial persistence and reduced treatment efficacy.

Some limitations should be considered. The sample size for certain staphylococcal species was limited, reducing the strength of species‐specific conclusions. In addition, the wide 95% confidence intervals observed, particularly for the prevalence of specific resistance profiles, indicate limited precision of the estimated effects and suggest a degree of model uncertainty due to these sample size constraints. Furthermore, the characterization of antimicrobial resistance profiles was based exclusively on phenotypic testing, and molecular analyses, such as PCR for the detection of *mec* genes (*mecA*/*mecC*) in methicillin‐resistant isolates, were not performed. While phenotypic testing represents the standard clinical workflow, this approach lacks the definitive accuracy of genotypic confirmation, carrying a minor risk of misclassifying borderline methicillin‐resistant strains. Finally, the lack of clinical history regarding prior antimicrobial treatments restricts the assessment of specific risk factors, and the cross‐sectional design precludes evaluating temporal relationships between colonisation and subsequent infection.

Overall, this study provides additional insights into the characteristics of staphylococci colonizing the nasal cavities of surgical canine patients. The detection of both biofilm‐producing and antimicrobial‐resistant strains highlights the complexity of the canine nasal microbiota and supports the role of these bacteria as potential reservoirs of resistance in veterinary environments. Further research incorporating larger populations, longitudinal designs, and molecular characterisation is warranted to fully clarify the epidemiological and clinical relevance of these findings.

## 5 Conclusion

Canine nasal cavities can harbour various *Staphylococcus* species, with *S. pseudintermedius* being the most prevalent. The high frequency of antimicrobial resistance, including the presence of MDR strains, together with the ability of these strains to form biofilms, underscores their potential clinical relevance and highlights the role of canine nasal staphylococci as reservoirs of antimicrobial resistance in veterinary settings. Consequently, preoperative nasal screening for MDR and biofilm‐forming staphylococci might be considered in high‐risk surgical canine patients to guide infection control measures and optimize prophylactic protocols.

## Author Contributions

**Francesca Paola Nocera**: methodology, investigation, data curation, formal analysis, writing – original draft, writing – review and editing, software, visualization. **Rossana Schena**: investigation, data curation, software, visualization. **Annunziata Romano**: investigation, software, visualization. **Stefano Cavalli**: investigation, visualization. **Laura Cortese**: writing – review and editing, visualization. **Gerardo Fatone**: methodology, writing – review and editing, visualization. **Luisa De Martino**: conceptualization, methodology, writing – original draft, writing – review and editing, funding acquisition, project administration, supervision, visualization.

## Funding

This study was funded by the European Union eNext Generation EU, PROGETTI DI RICERCA DI RILEVANTE INTERESSE NAZIONALE (PRIN 2022)—Occurrence and molecular analysis of methicillin‐resistant Staphylococci in cats and dogs: comparison between two Veterinary teaching Hospitals in North and South Italy (Project number: 2022TF32AL).

## Ethics Statement

This study was approved by the Institutional Animal Welfare Committee (OPBA, CSV, University of Naples Federico II, PG/2023/0120379 del 05/10/2023) in compliance with the Italian Legislative Decree 26/2014, Article 2, implementing Directive 2010/63/EU.

## Consent

Nasal swab samples were collected from household dogs undergoing orthopaedic surgery at the Veterinary Teaching Hospitals of the Department of Veterinary Medicine and Animal Production, University of Naples Federico II. All samples were obtained preoperatively by licensed veterinarians as part of routine clinical procedures, ensuring minimal discomfort and no additional risk to the animals. No invasive procedures were performed, and sample collection did not entail any additional risk beyond standard clinical handling. Written informed consent was obtained from the owners of the dogs prior to enrolment in the study.

## Conflicts of Interest

The authors declare no conflicts of interest.

## Data Availability

The data represent the findings of this study and are available within the article.
